# Brazilian consensus on vesicoureteral reflux–recommendations for clinical practice

**DOI:** 10.1590/S1677-5538.IBJU.2019.0401

**Published:** 2019-12-30

**Authors:** José Murillo B. Netto, Atila Victal Rondon, Marcos Giannetti Machado, Miguel Zerati, Rodrigo Lessa Pena Nascimento, Salvador Vilar Correa Lima, Adriano de Almeida Calado, Ubirajara Barroso

**Affiliations:** 1 Universidade Federal de Juiz de Fora Juiz de ForaMG Brasil Universidade Federal de Juiz de Fora -UFJF, Juiz de Fora , MG , Brasil ;; 2 Hospital e Maternidade Therezinha de Jesus Faculdade de Ciências Médicas e Saúde de Juiz de Fora Juiz de ForaMG Brasil Hospital e Maternidade Therezinha de Jesus da Faculdade de Ciências Médicas e Saúde de Juiz de Fora - HMTJ-SUPREMA, Juiz de Fora , MG , Brasil ;; 3 Universidade do Estado do Rio de Janeiro Rio de JaneiroRJ Brasil Universidade do Estado do Rio de Janeiro - UERJ, Rio de Janeiro , RJ , Brasil ;; 4 Hospital Federal Cardoso Fontes Rio de JaneiroRJ Brasil Hospital Federal Cardoso Fontes – HFCF, Rio de Janeiro , RJ , Brasil ;; 5 Universidade de São Paulo São PauloSP Brasil Universidade de São Paulo – USP, São Paulo , SP , Brasil ;; 6 Instituto de Urologia e Nefrologia de São José do Rio Preto Rio PretoSP Brasil Instituto de Urologia e Nefrologia de São José do Rio Preto – IUN, S. J. do Rio Preto , SP , Brasil ;; 7 Universidade Federal do Espírito Santo VitóriaES Brasil Universidade Federal do Espírito Santo – UFES, Vitória , ES , Brasil ;; 8 Universidade Federal de Pernambuco RecifePE Brasil Universidade Federal de Pernambuco (UFPE), Recife , PE , Brasil ;; 9 Hospital das Clínicas Faculdade de Medicina de Ribeirão Preto Universidade de São Paulo Ribeirão PretoSP Brasil Hospital das Clínicas da Faculdade de Medicina de Ribeirão Preto da Universidade de São Paulo - HCFMRP-USP, Ribeirão Preto , SP , Brasil ;; 10 Universidade Federal da Bahia SalvadorBA Brasil Universidade Federal da Bahia – UFBA, Salvador , BA , Brasil ;; 11 Escola Bahiana de Medicina SalvadorBA Brasil Escola Bahiana de Medicina – BAHIANA, Salvador , BA , Brasil

**Keywords:** Vesico-Ureteral Reflux, Urinary Tract Infections, Hydronephrosis

## Abstract

**Introduction:**

Vesicoureteral Reflux (VUR) is characterized by a retrograde flow of urine from the bladder into the ureters and kidneys. It is one of the most common urinary tract anomalies and the major cause of urinary tract infection (UTI) in the first years of life. If not properly diagnosed and treated can lead to recurrent UTI, renal scar and, in severe cases, to end stage renal disease. Despite recent advances in scientific and technological knowledge, evaluation and treatment of VUR is still controversial and there is still considerable heterogeneity in evaluation methods and therapeutic approaches. The aim of the present consensus is to give a practical orientation on how to evaluate and treat VUR.

**Methods:**

The board of Pediatric Urology of the Brazilian Society of Urology joined a group of experts and reviewed all important issues on Vesicoureteral Reflux evaluation and treatment and elaborated a draft of the document. On November 2017 the panel met to review, discuss and write a consensus document.

**Results and Discussion:**

Vesicoureteral Reflux is a common and challenging problem in children. Children presenting with Vesicoureteral Reflux require careful evaluation and treatment to avoid future urinary tract infections and kidney scars. The panel addressed recommendations on up to date choice of diagnosis evaluation and therapies.

## INTRODUCTION

Vesicoureteral reflux (VUR) is defined as the backflow of urine into the ureter and kidney. It is one of the most common urological anomalies in children with an incidence of 0.5% to 3% in the general pediatric population ( 1 , 2 ). This incidence increases to 30 to 40% in children with history of urinary tract infection (UTI) ( 3 , 4 ). The incidence of VUR in siblings of a child that has VUR varies from 26 to 46% ( 5 ).

The backflow of urine into the kidney predisposes bacteria to ascend causing pyelonephritis. The immunologic and inflammatory response to the infection may lead to renal lesions and formation of renal scars ( 6 , 7 ).

VUR is one of the most important diseases of childhood and, when not properly treated, presents high morbidity and can lead to significant renal damage and, if severe, consequent hypertension and chronic renal failure. Reflux nephropathy is responsible for up to 25% of cases of end stage renal disease ( 8 ).

The two most common forms of VUR presentation are urinary tract infection (UTI) and prenatal hydronephrosis. With the advent of antenatal ultrasound (US) more reflux cases are being diagnosed on the neonatal period. Of all cases of prenatal hydronephrosis, 15 to 21% are caused by VUR ( 9 , 10 ). Older children will mostly often be diagnosed after a febrile UTI.

**Figure 1:**
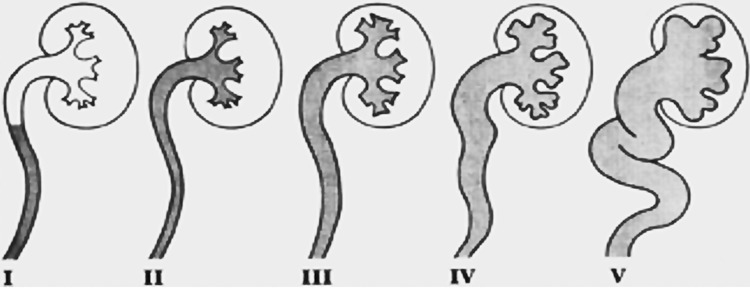
International Classification of VUR.

VUR is classified according the degree of ureteral, renal pelvis and calix dilation and varies according to severity from grade I to V ( Figure-1 ) ( 11 ). The use of a classification system is important to guide therapeutic approach, since lower grade VUR has a greater chance of spontaneous resolution and will benefit from more conservative treatments ( 12 ).

Investigation and management of VUR management is still controversial. Voiding Cystourethrography (VCUG) is considered the gold standard for diagnosing and evaluating VUR grade. Catheterization for VCUG can be traumatic for both the child and family ( 13 ). Not all children with UTI will present VUR, and of those with VUR, not all of them will present renal scar. Therefore, the indication of a VCUG for all children with prenatal hydronephrosis or UTI is debatable ( 14 - 17 ). Another important tool in the evaluation of VUR is the scintigraphy with DMSA (dimercaptosuccinic acid). DMSA scan is mostly used to investigate the impact of VUR in the kidney by analyzing function and the presence or not of renal scars. Debate whether it should be used in the acute phase of an UTI to rule out pyelonephritis and allow to avoid VCUG or in a later phase (4 to 6 months after UTI) to evaluate for scar formation is still debatable ( 18 ).

In the same way, the role of antibiotic prophylaxis and surgical treatment (endoscopic or ureteral reimplantation) have also been questioned and there is no clear indication of which the best treatment modality would be, especially in VUR of low or intermediate grades.

This Brazilian Guideline on evaluation and treatment of VUR has no intention to answer all these questions but to guide urologists, pediatricians, and pediatric nephrologists on the most recent aspects related to the management of children with vesicoureteral reflux.

## MATERIALS AND METHODS

The board of Pediatric Urology of the Brazilian Society of Urology, noticing the need of a Brazilian guideline on vesicoureteral reflux, joined a group of experts to review the important issues on VUR and elaborated a consensus document. Eight renewed pediatric urologist with known experience in dealing with urinary tract infections and vesicoureteral reflux were invited to participate in the elaboration of a document with the scope of the guiding urologists, pediatricians, nephrologists and others that deal with children with vesicoureteral reflux on the most important and up to date aspects of the evaluation and treatment of those children.

All panel members were instructed to perform a literature search on MEDLINE, EMBASE and COCHRANE LIBRARY databases as well as a review of the base of practical guidelines database for the last 20 years using the term “vesicoureteral reflux”. Papers were selected according to their level of evidence, giving more importance to meta-analysis, systematic reviews, and randomized controlled trials. Cohort and series of patients were used to add information. Review papers and guidelines were used as orientation for which topics and aspects would be included.

After the papers were selected, each member of the group was designated one topic to review and write an orientation document based on the recommended literature.

On November 2017, all members joined together during 2 days to review and discuss the previous written documents of each topic and prepare the consensus document. Further discussions, corrections, and revisions were carried out digitally, until all members of the panel have approved this final document. A paragraph containing the panels opinion (“consensus”) was added at the end of each section to guide the reader about the information provided and the most common practice on each specific subject.

## CLINICAL EVALUATION AND DIAGNOSIS (Figure-2)

As in all fields of medicine, a careful clinical history is very important for the diagnosis. Aspects related to the presence of prenatal hydronephrosis, past episodes of febrile and non-febrile UTI should be investigated. Understanding voiding and bowel habits are important since lower urinary tract dysfunction (LUTD) and constipation are often associated with UTI and VUR ( 19 - 21 ). VUR diagnosed in the neonatal period is more common in boys and of a higher grade ( 22 ) and is related to high bladder pressure and post-voided residual urine ( 23 ). High bladder pressure in infancy may predispose or difficult spontaneous VUR resolution ( 21 , 23 ).

**Figure 2:**
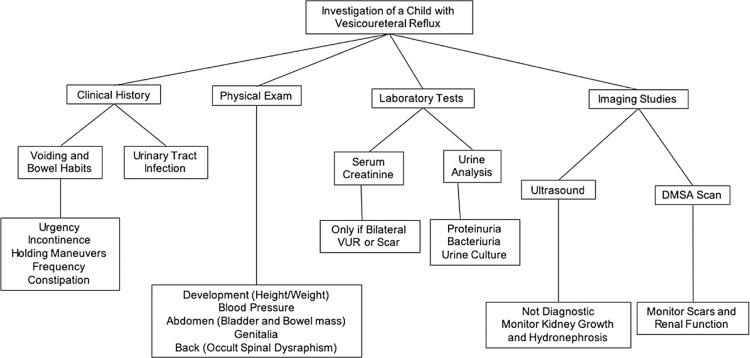
Clinical investigation of a child presenting VUR.

In all toilet trained children, a very meticulous clinical history of their voiding symptoms, such as increased voiding frequency, incontinence, urinary urgency, holding maneuvers, and also, constipation should be taken. Physical examination should include assessment of weight, height, and blood pressure, palpation of the abdomen looking for masses and globus vesicalis, presence of feces in the bowel, and evaluation of the genitalia. Examination of the back in search for skin markers suggesting occult spinal dysraphism is important since VUR is present in up to 25% of children with spinal dysraphism ( 24 ).

Clinical history should be periodically reevaluated during follow-up, since symptoms may change. LUTD and constipation should also be frequently assessed during the course of treatment.

The final diagnosis of VUR will be obtained only with an imaging test. The imaging test for defining VUR diagnosis should be ideally radiation free, with no need for urethral catheterization or sedation, presenting high accuracy and anatomical detailing and with low cost. Unfortunately, none of the currently available imaging tests (VCUG or direct cystocinthigraphy) fills all or most parameters named before.

### Laboratory Tests

Serum creatinine dosage is indicated in cases of bilateral high grade VUR and/or presence of bilateral renal scars, being a parameter to estimate the rate of glomerular filtration and as a baseline for future comparisons.

Urine analysis, including proteinuria, bacteriuria, and urine culture are recommended for the diagnosis of VUR and subsequently for suspected UTI. The recommended method for urine collection in children that are not yet toilet-trained is via clean urethral catheterization to avoid contamination ( 25 , 26 ).

We do not recommend periodic urine analysis and urine culture in asymptomatic children. Investigation of UTI in cases of fever of undetermined origin in patients with VUR must always be performed.

## IMAGING STUDIES

### Ultrasound

Ultrasound is not accurate in predicting the presence of VUR and should not be used for the diagnosis of VUR ( 27 - 29 ).

Ultrasonography of the urinary tract is recommended to monitor renal development, as well as assess the occurrence or worsening of hydronephrosis, and presence of post voided residue urine. It is important to observe bladder filling during the exam, as this may be correlated with the degree of renal dilation. Ultrasound examinations should be performed at least every 6 months.

### Renal Scan

The goals of DMSA scan are to look for the appearance or progression of renal scars and monitoring renal function ( 30 , 31 ). The best time to order a DMSA scan for the evaluation of VUR is still debatable. Two different approaches have been proposed with the DMSA scan done either in the acute phase of an UTI episode or after 6 months post-infection ( 25 , 32 , 33 ).

The “top-down” approach, which means that the evaluation starts from the kidney by ordering the DMSA scan during the acute phase of the UTI was proposed with the aim to avoid unnecessary VCUG and has a sensitivity of up to 95% ( 34 ). In this approach VCUG is only ordered in those with an abnormal DMSA scan. A problem regarding this “top-down approach” is that a second DMSA scan may be needed after 6 months of the UTI to evaluate scar formation.

On the contrary, the “bottom-down” approach ( 25 ) advices that the DMSA scan should only be performed 6 months after the UTI with the main goal to evaluate the presence of permanent scars.

In a less invasive way of evaluating children with UTI DMSA, scan would be ordered only in cases of febrile UTI, high grade VUR (IV and V), and changes on ultrasound suggestive of renal lesions.

As for periodicity, DMSA scans should be repeated only after presentation of new episodes of febrile UTI.

### Voiding Cystourethrography (VCUG)

Voiding Cystourethrography (VCUG) uses iodine as a contrast medium and allows the classification of VUR as well as evaluation of bladder and urethral anatomy. Because reflux may be an intermittent phenomenon, the test should be performed with fluoroscopic monitoring and with more than one bladder filling cycle, not to exceed three cycles.

It is recommended that it should be done at earliest convenience following UTI treatment ( 35 ), confirmation of a sterile urine and with antibiotic coverage due to the risk of onset of a new episode of UTI ( 36 ).

The main advantage of VCUG over Direct Isotopic Radionuclide Cystography is related to the anatomical detail. In addition, the current VUR grading system is based on VCUG. Therefore, VCUG remains the gold standard diagnostic test and initial evaluation of VUR.

### Direct Isotopic Radionuclide Cystography (DIRC)

Direct Isotope Radionuclide Cystography can replace VCUG for the diagnosis or follow-up of patients with VUR. In this method, a radio-isotopic tracer (usually diethyltriaminepentaacetic acid-DTPA) is infused in the bladder after urethral catheterization and images are obtained during bladder filling and emptying.

Although radio-isotopic method is believed to have less radiation exposure ( 3 ), a recent study demonstrated higher radiation exposure compared to fluoroscopic cystography ( 37 ). A good correlation was seen between DIRC and VCUG in diagnosing VUR ( 38 ) although DIRC has the disadvantage of low definition of image, not allowing the anatomical evaluation of the bladder and urethra, nor proper VUR classification ( 3 ). The use of DIRC is preferred during clinical follow-up or evaluation of surgical treatment result.

### Other exams in the diagnosis of VUR

Other methods have been developed in an attempt to reduce the morbidity of traditional exams (VCUG and DIRC) in the diagnosis of VUR. Ultrasonographic Cystography has been shown to be very accurate in diagnosing VUR ( 39 , 40 ) although its use is not yet widespread. Indirect Magnetic Resonance Cystography although is an option to avoid radiation and catheterization, it has been shown to be less sensitive than VCUG in diagnosing lower grade VUR and with higher cost ( 41 , 13 ).

### Consensus

The panel believes that a careful and meticulous clinical history considering all aspects discussed above and with special attention to LUTD should be obtained prior to any imaging test. All children should be evaluated with a renal ultrasound with the evaluation of post-voided residual urine. Renal Scans with DMSA should be reserved for those with history of febrile UTI, VUR grade IV or V and ultrasound suggesting renal lesions. VCUG should be the imaging test of choice for the diagnosis of VUR. DIRC should only be indicated on the follow-up, especially after surgical treatment.

## WHO WILL BENEFIT FROM INVESTIGATION

The indication for VCUG may vary according to the clinical presentation of the patient and some protocols have been proposed for this purpose.

### Children with urinary tract infection

The indication of a VCUG in the evaluation of a child presenting UTI is still controversial. Children presenting febrile recurrent ITU and/or in cases where alterations of the urinary tract are found in the ultrasonography should be evaluated with a VCUG ( 25 ).

Despite that requesting a VCUG after the first episode of febrile UTI in infants is still questioned by some authors, we believe that it could be done in those cases ( 1 ).

On the other hand, in older children with recurrent afebrile UTI, VCUG is exceptionally indicated, since the main etiology of UTI in this group of patient is LUTD ( 42 ).

### Children with Antenatal Hydronephrosis

VCUG is recommended in newborns with postnatal ultrasound findings of bilateral grade II to IV and unilateral grade III to IV hydronephrosis-Society of Fetal Urology-SFU ( 43 , 44 ), signs of duplicity with hydronephrosis, ureterocele, ureteral dilatation and vesical changes.

For grade II hydronephrosis its indication is controversial, but there may be benefits. In case of degree I hydronephrosis its routine indication may be dispensable.

### Siblings and Children of Patients with History of VUR

Routine investigation of asymptomatic siblings and/or children of patients with VUR is controversial. The lack of randomized clinical trials to detect VUR in these patients makes it difficult to routinely recommend it. Parents of children with VUR must be informed that there is a high prevalence of reflux in siblings and offspring, and if the decision is made to investigate, the initial examination should be ultrasonography, with VCUG reserved only for cases of significant changes on ultrasound or after UTI episodes ( 45 , 46 ).

### Consensus

Although the indications for investigation of VUR in children presenting UTI are controversial, the panel agrees that is mandatory that all children with febrile UTI and changes in the ultrasound, and infants with UTI, regardless of changes in US, must be investigated, and encourages investigation of children with well documented UTI, regardless of changes in US. Older children should be carefully evaluated for LUTD. Children presenting with prenatal hydronephrosis should only be routinely investigated if they present high-grade hydronephrosis (grades III and IV) or if ureteral dilation. Investigation of siblings and offspring of patients with VUR should be discussed with the family and, if investigation is the option, it should start with US.

## CONTINUOUS ANTIBIOTICS PROPHYLAXIS (CAP)

The use of low-dose antibiotics to prevent UTI in children with VUR is based on the observation that VUR has a high spontaneous resolution rate in the first 4 to 5 years of life (80% grade III VUR, 30-50% grades III-IV) ( 47 - 50 ) and has been indicated for more than 4 decades. This clinical practice is based mainly on expert opinions and, until recently, with few randomized and controlled trials ( 51 - 53 ). Since the 2000s, better quality studies have begun to question whether CAP actually protects children with VUR from pyelonephritis and the formation of new renal scars and if there is a specific group of children who would benefit most from this practice ( 54 - 57 ).

Recently, a large multicenter, randomized study including 607 children with VUR diagnosed after the first or second UTI and with a 2-year clinical follow-up demonstrated that CAP is associated with a significant reduction in the risk of UTI episodes but not new scars (Grade of Recommendation A) ( 58 ). Recent meta-analysis have demonstrated benefits of CAP in infants with all degrees of VUR ( 59 - 62 ).

The duration of CAP is still controversial. One option would be to perform VCUG periodically (intervals of not less than 1 year) and, if there is resolution of the reflux, stop the CAP. Another option is stop CAP in toilet trained children with no LUTD. In children who, even when using CAP, present new episodes of UTI, surgical treatment should be an option ( 2 ).

### Types of Medications Used in Reflux Antibiotics Prophylaxis

Continuous antibiotic prophylaxis, when instituted, should be adequate for the child’s age group and the antimicrobial susceptibility pattern of the population in the area the child lives.

The drug of choice should be well tolerated, with low risks and side effects and be affordable, considering ongoing treatment. The dose to be administered is between 25 to 50% of the therapeutic dose, which should be adjusted periodically, according to the child’s weight gain, which is more significant in the first year of life. The drug of choice in infants, in the first 6 months of life, by the availability and drug safety, should be Cephalexin or Amoxicillin. Use of Sulfamethoxazole and Nitrofurantoin are not indicated before 2 months of age. For children older than 6 months of age, the options would be Cephalexin, Amoxicillin, Sulfamethoxazole/Trimethoprim, Nitrofurantoin or Nalidixic Acid.

### Consensus

Based on the studies discussed above, the recommendation of this panel is that CAP should be indicated in all infants and children who have not yet completed sphincter training and who present VUR grade III or higher. However, those with VUR grade I and II also appear to benefit from CAP and the decision should be made after discussing with the family.

## FACTORS RELATED TO SPONTANEOUS RESOLUTION OF VUR (Figure-3)

The management of VUR aims to prevent the onset of new episodes of UTI and loss of renal function. Clinical treatment consists of continuous administration of low-dose antibiotics to maintain sterile urine and thereby prevent pyelonephritis and formation of renal scars. The basis of clinical treatment is the expectation of spontaneous resolution, since VUR tends to decrease in grade or completely resolve with time ( 48 , 50 ). The identification of factors that predict spontaneous VUR resolution may contribute to family counseling at the time of diagnosis and assist in the choice of treatment strategies.

**Figure 3:**
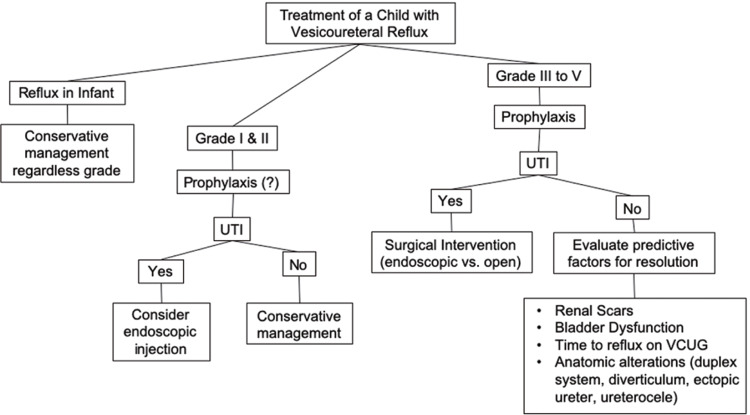
Management of VUR.

### Main factors that predicts spontaneous resolution are

**Grade of VUR:** The higher the grade of the VUR, the lower the chances of spontaneous resolution. Refluxes of dilated degrees (IV and V) present a probability of spontaneous resolution of 5 to 20%, while in VUR grades I and II resolution occurs in more than 80% ( 48 , 50 , 63 , 64 ).

**Age at Presentation:** VUR presenting in postnatal evaluation or before 1 year of age are associated with earlier resolution ( 50 , 65 , 66 ).

**Gender:** Boys with VUR tend to present spontaneous resolution prior to girls ( 67 ).

**Laterality:** Bilateral high-grade VUR (III to V) presents a lower probability of spontaneous resolution compared to unilateral VUR ( 50 , 67 ).

**Abnormalities on DMSA:** When renal scars or functional deficit are present there will be lower chances of spontaneous resolution of VUR ( 1 , 68 , 69 ).

**Infused Volume at Presentation of VUR on VCUG:** Refluxes that appear in the early stages of bladder filling present smaller possibilities of spontaneous resolution, whereas refluxes that appear only during urination present higher resolution rates ( 63 , 64 , 70 ).

**Urinary Tract Infection:** The development of an UTI episode during clinical follow-up is a negative predictor for VUR resolution ( 71 ) and a sign that clinical approach should be reviewed and an alternative intervention may be required ( 44 ).

**Bowel and Bladder Dysfunction:** The presence of LUTD and/or constipation has a negative impact on VUR resolution.

**Diameter of the Distal Ureter:** The diameter of the distal ureter is an independent predictor of spontaneous resolution of VUR. As smaller is the diameter of the distal ureter, the greater the chance of spontaneous resolution ( 47 ).

**Associated Anomalies:** The presence of pyelo-ureteral duplicity or para-ureteral diverticulum are some of the anatomical factors related to reduction of spontaneous resolution ( 50 , 64 , 70 ).

### Consensus

The panels opinion is that all the above mentioned factors should be evaluated and taken into consideration when discussing with the family the therapeutic options for treating a child with VUR. This panel strongly recommends that treatment of LUTD and constipation should precede any intervention for treatment of VUR ( 1 , 68 , 72 ). The use of nomograms and calculators may be helpful in the evaluation of the chances of a new breakthrough UTI ( 73 ) and of spontaneous resolution of the VUR ( 64 , 70 ).

## SURGICAL RECOMMENDATIONS FOR THE TREATMENT OF VUR (Figure-3)

There is a lack of prospective studies with a control group to establish a safe guideline for VUR treatment. Thus, it is not possible to produce recommendations with a high level of evidence.

The objective of VUR treatment is minimizing the risk of pyelonephritis and preventing the risk for development of new renal scars with the ultimate goal of preventing renal failure ( 44 ). It is based on the risk factors of each patient, such as age, sex, grade of VUR, and presence of LUTD, breakthrough UTI, anatomical abnormalities and renal status. Patients at high risk for developing UTI or renal scars should be carefully managed.

However, the controversies persist regarding the best treatment of VUR, particularly in the choice between observation alone, CAP, endoscopic treatment or ureteral reimplantation, and, if surgical treatment is indicated, best time to perform it.

Surgical recommendations can be divided in absolute and relative indications. Absolute Recommendations include repeated UTI despite CAP, VUR that have low chance of spontaneous resolution, and preference of the parents ( 63 , 74 - 76 ). After discussing the risks and possible outcomes with the parents, surgery should be considered if it is their will, regardless of whether it would be endoscopic injection or ureteral reimplantation. Relative Recommendations are persistence of VUR grade III to V in asymptomatic patients; presence of renal scaring, VUR grades III to V in patients with renal scars, children with difficulty to maintain clinical follow-up and to have access to health services, persistence of VUR in girls after the age of 5 years ( 1 , 44 , 75 , 77 - 79 ).

### Circumcision

Circumcision for children with VUR has been shown to reduce the frequency of positive urine culture although no difference was found in symptomatic UTI and changes in DMSA scan when compared to no circumcision. Its indications in children with VUR reflux should be discussed with the family.

### High Grade VUR in Neonates

Severe VUR in neonates may be seen with caution. In up to 59% of cases it will improve or spontaneously resolve and should be initially managed with CAP ( 80 , 81 ). Those with end stage renal disease or presenting pyelonephritis may need early surgical intervention. Options include vesicostomy, pyelostomy, and ureterostomy.

### Endoscopic Treatment for VUR

Endoscopic injection of a bulking agent is the least invasive procedure for treating VUR ( 82 ) that can be indicated even before completing 1 year of life ( 83 ). There is evidence that endoscopic treatment reduces the rate of UTI compared to observation, but it is similar to CAP in short term follow-up, but presents a higher cure rate when compared to observation alone ( 71 , 84 ). On the other hand, its success rate is lower than open surgery (ureteral reimplantantion), specially for high grade VUR ( 85 ).

### Bulking Agents

Polymethylsiloxane (Macroplastique®): Non-absorbable. Due to the greater hardness of the material, it is necessary to use an injection gun ( 86 - 88 ).

Dextranomer/Hyaluronic Acid (Deflux®): Advantage of being easy to inject and with fewer complications ( 89 ). As a disadvantage, it is partially absorbed, causing loss of some volume in the long term, with recurrence of VUR in about 20% of the cases ( 90 ).

Polyalcohol/Polyacrylate (Vantris®): Not to be absorbed and easy to inject. As a disadvantage, it causes a higher inflammatory process and, therefore, has a higher risk for obstruction ( 91 - 93 ).

Pyrolytic Carbon (Durasphere®): Its application is difficult and there are few studies showing its effectiveness ( 94 ).

### Endoscopic Treatment Technique

Subureteral injection (STING): In this technique, the injection site is about 2-3mm below the ureter orifice (at 6 o’clock) and the needle is deepened by 4-5mm ( 95 , 96 ).

Hydrodistension Injection Technique (HIT): In this technique, the flow of endoscopic irrigation is positioned immediately in front of the ureteral meatus. The substance is injected approximately 5mm into the ureter. More than one injection is possible with this technique (Double HIT) ( 96 , 97 , 98 ).

### Success rate

The higher the VUR grade the lower the success rate. Other factors related to lower success rate are LUTD, surgeon’s experience, and previous injection ( 75 , 99 - 101 ).

### Postoperative follow-up

Patients should perform ultrasonography after surgery, preferably between one and three months ( 90 , 92 , 102 ).

Performing VCUG after the procedure is optional, and should be indicated in case of relapse of febrile UTI.

### Consensus

This panel recommends the endoscopic treatment of VUR as the first surgical treatment option, except for Grade V VUR with significant ureteral dilatation.

The panel also recommends that after a second unsuccessful endoscopic injection, the possibility of treatment with open surgery should be considered. There is insufficient data in the literature to evaluate the results of re-application of Polyalcohol/Polyacrylate. Therefore, according to this panel, open surgery should be considered after failure to a first injection with this material.

If Dextranomer/Hyaluronic Acid is the bulking agent of choice, consider injecting higher volumes and use of HIT technique. If Polyalcohol/Polyacrylate is the bulking agent chosen, it is advised to use lower volumes and not use the HIT technique due to the higher risk of obstruction.

This panel recommends performing at least one annual ultrasonography, as late obstructions have been reported, especially after Polyalcohol/Polyacrylate injection.

### Open surgery

Ureteral reimplantation is the most effective approach to prevent new episodes of febrile UTI, especially in high grade VUR or after unsuccessful endoscopic injection. All techniques have high success rates (>95%) ( 44 , 75 ).

Complications include the possibility of obstruction (2%) and contralateral reflux (9%).

The principle of all ureteral reimplantation techniques is to create a longer submucosal tunnel, four to five times the diameter of the ureter, in an attempt to reproduce the physiological anti-reflux mechanism of compressing the ureter as intra-vesical pressure increases with filling and urination ( 103 ).

Intra and extra-vesical procedures as well as combined techniques have been described. The choice of technique depends on the degree of dilation of the ureter, whether the reflux is unilateral or bilateral, the presence of other obstructions, and the preference of the surgeon.

### The most used techniques are: Extra-Vesical: Lich-Gregoir (104).

**Intra-Vesical:** Cohen and Glenn-Anderson ( 105 ) and Politano-Leadbetter ( 106 ).

Bilateral extra-vesical techniques may present an increased risk of postoperative transient bladder dysfunction and urinary retention ( 107 ). In cases of unilateral VUR, the preference is for the extra-vesical approach (Litch-Gregoir technique) ( 2 , 108 ).

Cohen’s intra-vesical technique consists of bilateral crossing ureteral reimplantation, with the construction of a long tunnel, with a low risk of obstruction by ureter angulation. However, there is the disadvantage of possibly hindering retrograde endoscopic procedures in the future ( 2 , 108 , 109 ).

The combined technique of Politano-Leadbetter allows the construction of a longer tunnel, being very useful in reimplantation of a dilated ureter, but with a slightly greater risk of obstruction by angulation of the ureter. The meatus is positioned in an easily accessible position for endoscopic manipulation ( 2 , 110 ).

The Glenn-Anderson technique, with intra-vesical advancement of the ureter towards the bladder neck, has a low risk of ureter angle obstruction, but presents a limit to the length of the tunnel ( 2 ).

### Laparoscopic/Robotics Surgery

Laparoscopic and robotic techniques present long learning curve, even for experienced surgeons, with long operative times than open procedures. Nowadays, success rates are as high as open surgery with few complications ( 111 - 113 ). The main disadvantage is the cost, which is higher than any other treatment modality.

### Consensus

It is the panels opinion that high grade VUR (grade V and some cases of grade IV) should be treated with ureteral reimplantation, either with open of laparoscopic/robotic techniques depending on the experience of the surgeon and the availability of the technology. In unilateral cases, extra-vesical approach should be considered while in bilateral cases, intra-vesical technique (Cohen) would be preferable.

## POST-OPERATIVE FOLLOW-UP

There is no consensus regarding postoperative follow-up both in endoscopic treatment and in open, laparoscopic or robotic surgery. As the success rate of the procedures is high, it is not recommended, in general, to perform control VCUG in all patients, which should be indicated in patients with new episodes of febrile UTI and, possibly, in patients with high grade VUR treated with endoscopic procedure, where the success rate is lower.

Ultrasonography is performed between 1 and 3 months after the surgical procedure and is performed at regular intervals after endoscopic treatment because of the risk of late obstruction.

### Consensus

It is the panel’s opinion that a kidney and bladder ultrasound should be done after the first month of surgery to check for signs of obstruction. VCUG is indicated only in case of breakthrough UTI or after endoscopic treatment of high grade VUR.

## References

[1] 1. Arlen AM , Cooper CS . Controversies in the Management of Vesicoureteral Reflux . Curr Urol Rep . 2015 ; 16 : 64 . 10.1007/s11934-015-0538-2 26199037

[2] 2. Hajiyev P , Burgu B . Contemporary Management of Vesicoureteral Reflux . Eur Urol Focus . 2017 ; 3 : 181 - 188 . 10.1016/j.euf.2017.08.012 28918954

[3] 3. Lebowitz RL . The detection and characterization of vesicoureteral reflux in the child . J Urol . 1992 ; 148 ( 5 Pt 2 ): 1640 - 2 . 10.1016/s0022-5347(17)36991-4 1433579

[4] 4. Sargent MA . What is the normal prevalence of vesicoureteral reflux? Pediatr Radiol . 2000 ; 30 : 587 - 93 . 10.1007/s002470000263 11009294

[5] 5. Kenda RB , Kenig T , Budihna N . Detecting vesico-ureteral reflux in asymptomatic siblings of children with reflux by direct radionuclide cystography . Eur J Pediatr . 1991 ; 150 : 735 - 7 . 10.1007/BF01958768 1915489

[6] 6. Ransley PG , Risdon RA . The pathogenesis of reflux nephropathy . Contrib Nephrol . 1979 ; 16 : 90 - 7 . 10.1159/000402880 467074

[7] 7. Pokrajac D , Sefic-Pasic I , Begic A . Vesicoureteral Reflux and Renal Scarring in Infants After the First Febrile Urinary Tract Infection . Med Arch . 2018 ; 72 : 272 - 5 . 10.5455/medarh.2018.72.272-275 PMC6195033 30514993

[8] 8. Gusmano R , Perfumo F . Worldwide demographic aspects of chronic renal failure in children . Kidney Int Suppl . 1993 ; 41 : S31 - 5 . 8320942

[9] 9. Phan V , Traubici J , Hershenfield B , Stephens D , Rosenblum ND , Geary DF . Vesicoureteral reflux in infants with isolated antenatal hydronephrosis . Pediatr Nephrol . 2003 ; 18 : 1224 - 8 . 10.1007/s00467-003-1287-x 14586679

[10] 10. Brophy MM , Austin PF , Yan Y , Coplen DE . Vesicoureteral reflux and clinical outcomes in infants with prenatally detected hydronephrosis . J Urol . 2002 ; 168 ( 4 Pt 2 ): 1716 - 9 ; discussion 1719 . 10.1097/01.ju.0000026907.65728.6e 12352342

[11] 11. Lebowitz RL , Olbing H , Parkkulainen KV , Smellie JM , Tamminen-Möbius TE . International system of radiographic grading of vesicoureteric reflux. International Reflux Study in Children . Pediatr Radiol . 1985 ; 15 : 105 - 9 . 10.1007/BF02388714 3975102

[12] 12. Zerati Filho M , Calado AA , Barroso U Jr , Amaro JL . Spontaneous resolution rates of vesicoureteral reflux in Brazilian children: a 30-year experience . Int Braz J Urol . 2007 ; 33 : 204 - 12 ; discussion 213-5 . 10.1590/s1677-55382007000200012 17488541

[13] 13. Johnin K , Kobayashi K , Tsuru T , Yoshida T , Kageyama S , Kawauchi A . Pediatric voiding cystourethrography: An essential examination for urologists but a terrible experience for children . Int J Urol . 2019 ; 26 : 160 - 71 . 10.1111/iju.13881 30569659

[14] 14. Newman TB . The new American Academy of Pediatrics urinary tract infection guideline . Pediatrics . 2011 ; 128 : 572 - 5 . 10.1542/peds.2011-1818 21873698

[15] 15. Lee LC , Lorenzo AJ , Odeh R , Falkiner M , Lebarron DA , Traubici J , et al . Contemporary Practice Patterns of Voiding Cystourethrography Use at a Large Tertiary Care Center in a Single Payer Health Care System . J Urol . 2017 ; 197 ( 3 Pt 2 ): 951 - 6 . 10.1016/j.juro.2016.08.102 27593475

[16] 16. Garcia-Roig M , Travers C , McCracken CE , Kirsch AJ . National Trends in the Management of Primary Vesicoureteral Reflux in Children . J Urol . 2018 ; 199 : 287 - 93 . 10.1016/j.juro.2017.09.073 28941917

[17] 17. Nguyen HT , Herndon CD , Cooper C , Gatti J , Kirsch A , Kokorowski P , et al . The Society for Fetal Urology consensus statement on the evaluation and management of antenatal hydronephrosis . J Pediatr Urol . 2010 ; 6 : 212 - 31 . 10.1016/j.jpurol.2010.02.205 20399145

[18] 18. La Scola C , De Mutiis C , Hewitt IK , Puccio G , Toffolo A , Zucchetta P , et al . Different guidelines for imaging after first UTI in febrile infants: yield, cost, and radiation . Pediatrics . 2013 ; 131 : e665 - 71 . 10.1542/peds.2012-0164 23439905

[19] 19. Sillén U . Bladder dysfunction in children with vesico-ureteric reflux . Acta Paediatr Suppl . 1999 ; 88 : 40 - 7 . 10.1111/j.1651-2227.1999.tb01318.x 10588270

[20] 20. Koff SA . Relationship between dysfunctional voiding and reflux . J Urol . 1992 ; 148 ( 5 Pt 2 ): 1703 - 5 . 10.1016/s0022-5347(17)37007-6 1433592

[21] 21. Ural Z , Ulman I , Avanoglu A . Bladder dynamics and vesicoureteral reflux: actors associated with idiopathic lower urinary tract dysfunction in children . J Urol . 2008 ; 179 : 1564 - 7 . 10.1016/j.juro.2007.11.095 18295262

[22] 22. Yeung CK , Godley ML , Dhillon HK , Gordon I , Duffy PG , Ransley PG . The characteristics of primary vesico-ureteric reflux in male and female infants with pre-natal hydronephrosis . Br J Urol . 1997 ; 80 : 319 - 27 . 10.1046/j.1464-410x.1997.00309.x 9284209

[23] 23. Sjöström S , Bachelard M , Sixt R , Sillén U . Change of urodynamic patterns in infants with dilating vesicoureteral reflux: 3-year followup . J Urol . 2009 ; 182 : 2446 - 53 . 10.1016/j.juro.2009.07.057 19765771

[24] 24. Netto JM , Bastos AN , Figueiredo AA , Pérez LM . Spinal dysraphism: a neurosurgical review for the urologist . Rev Urol . 2009 ; 11 : 71 - 81 . PMC2725308 19680528

[25] 25. Subcommittee on Urinary Tract Infection , Steering Committee on Quality Improvement and Management , Roberts KB . Urinary tract infection: clinical practice guideline for the diagnosis and management of the initial UTI in febrile infants and children 2 to 24 months . Pediatrics . 2011 ; 128 : 595 - 610 . 10.1542/peds.2011-1330 21873693

[26] 26. Ammenti A , Cataldi L , Chimenz R , Fanos V , La Manna A , Marra G , et al . Febrile urinary tract infections in young children: recommendations for the diagnosis, treatment and follow-up . Acta Paediatr . 2012 ; 101 : 451 - 7 . 10.1111/j.1651-2227.2011.02549.x 22122295

[27] 27. Nelson CP , Johnson EK , Logvinenko T , Chow JS . Ultrasound as a screening test for genitourinary anomalies in children with UTI . Pediatrics . 2014 ; 133 : e394 - 403 . 10.1542/peds.2013-2109 PMC3934332 24515519

[28] 28. Logvinenko T , Chow JS , Nelson CP . Predictive value of specific ultrasound findings when used as a screening test for abnormalities on VCUG . J Pediatr Urol . 2015 ; 11 : 176.e1 - 7 . 10.1016/j.jpurol.2015.03.006 PMC4540607 25958031

[29] 29. Mahant S , Friedman J , MacArthur C . Renal ultrasound findings and vesicoureteral reflux in children hospitalised with urinary tract infection . Arch Dis Child . 2002 ; 86 : 419 - 20 . 10.1136/adc.86.6.419 PMC1762998 12023172

[30] 30. Montini G , Zucchetta P , Tomasi L , Talenti E , Rigamonti W , Picco G , et al . Value of imaging studies after a first febrile urinary tract infection in young children: data from Italian renal infection study 1 . Pediatrics . 2009 ; 123 : e239 - 46 . 10.1542/peds.2008-1003 19139086

[31] 31. Roupakias S , Sinopidis X , Tsikopoulos G , Spyridakis I , Karatza A , Varvarigou A . Dimercaptosuccinic acid scan challenges in childhood urinary tract infection, vesicoureteral reflux and renal scarring investigation and management . Minerva Urol Nefrol . 2017 ; 69 : 144 - 52 . 10.23736/S0393-2249.16.02509-1 27355216

[32] 32. Hansson S , Dhamey M , Sigström O , Sixt R , Stokland E , Wennerström M , et al . Dimercapto-succinic acid scintigraphy instead of voiding cystourethrography for infants with urinary tract infection . J Urol . 2004 ; 172 : 1071 - 3 . 10.1097/01.ju.0000135337.71154.60 15311040

[33] 33. Preda I , Jodal U , Sixt R , Stokland E , Hansson S . Normal dimercaptosuccinic acid scintigraphy makes voiding cystourethrography unnecessary after urinary tract infection . J Pediatr . 2007 ; 151 : 581 - 4 . 10.1016/j.jpeds.2007.05.008 18035134

[34] 34. Herz D , Merguerian P , McQuiston L , Danielson C , Gheen M , Brenfleck L . 5-year prospective results of dimercapto-succinic acid imaging in children with febrile urinary tract infection: proof that the top-down approach works . J Urol . 2010 ; 184 ( 4 Suppl ): 1703 - 9 . 10.1016/j.juro.2010.04.050 20728131

[35] 35. Koyle MA , Elder JS , Skoog SJ , Mattoo TK , Pohl HG , Reddy PP , et al . Febrile urinary tract infection, vesicoureteral reflux, and renal scarring: current controversies in approach to evaluation . Pediatr Surg Int . 2011 ; 27 : 337 - 46 . 10.1007/s00383-011-2863-y 21305381

[36] 36. Rachmiel M , Aladjem M , Starinsky R , Strauss S , Villa Y , Goldman M . Symptomatic urinary tract infections following voiding cystourethrography . Pediatr Nephrol . 2005 ; 20 : 1449 - 52 . 10.1007/s00467-005-1942-5 16047224

[37] 37. Haid B , Becker T , Koen M , Berger C , Langsteger W , Gruy B , et al . Lower radiation burden in state of the art fluoroscopic cystography compared to direct isotope cystography in children . J Pediatr Urol . 2015 ; 11 : 35.e1 - 6 . 10.1016/j.jpurol.2014.08.015 25748630

[38] 38. Unver T , Alpay H , Biyikli NK , Ones T . Comparison of direct radionuclide cystography and voiding cystourethrography in detecting vesicoureteral reflux . Pediatr Int . 2006 ; 48 : 287 - 91 . 10.1111/j.1442-200X.2006.02206.x 16732797

[39] 39. Piscitelli A , Galiano R , Serrao F , Concolino D , Vitale R , D’Ambrosio G , et al . Which cystography in the diagnosis and grading of vesicoureteral reflux? Pediatr Nephrol . 2008 ; 23 : 107 - 10 . 10.1007/s00467-007-0651-7 17987321

[40] 40. Darge K . Voiding urosonography with US contrast agent for the diagnosis of vesicoureteric reflux in children: an update . Pediatr Radiol . 2010 ; 40 : 956 - 62 . 10.1007/s00247-010-1623-9 20432014

[41] 41. Lee SK , Chang Y , Park NH , Kim YH , Woo S . Magnetic resonance voiding cystography in the diagnosis of vesicoureteral reflux: comparative study with voiding cystourethrography . J Magn Reson Imaging . 2005 ; 21 : 406 - 14 . 10.1002/jmri.20273 15779038

[42] 42. Barroso U Jr , Barroso DV , Jacobino M , Vinhaes AJ , Macedo A Jr , Srougi M . Etiology of urinary tract infection in scholar children . Int Braz J Urol . 2003 ; 29 : 450 - 4 . 10.1590/s1677-55382003000500012 15745593

[43] 43. Nguyen HT , Benson CB , Bromley B , Campbell JB , Chow J , Coleman B , et al . Multidisciplinary consensus on the classification of prenatal and postnatal urinary tract dilation (UTD classification system) . J Pediatr Urol . 2014 ; 10 : 982 - 98 . 10.1016/j.jpurol.2014.10.002 25435247

[44] 44. Peters CA , Skoog SJ , Arant BS Jr , Copp HL , Elder JS , Hudson RG , et al . Summary of the AUA Guideline on Management of Primary Vesicoureteral Reflux in Children . J Urol . 2010 ; 184 : 1134 - 44 . 10.1016/j.juro.2010.05.065 20650499

[45] 45. Skoog SJ , Peters CA , Arant BS Jr , Copp HL , Elder JS , Hudson RG , et al . Pediatric Vesicoureteral Reflux Guidelines Panel Summary Report: Clinical Practice Guidelines for Screening Siblings of Children With Vesicoureteral Reflux and Neonates/Infants With Prenatal Hydronephrosis . J Urol . 2010 ; 184 : 1145 - 51 . Erratum in: J Urol. 2011;185:365 . 10.1016/j.juro.2010.05.066 20650494

[46] 46. Nelson CP , Finkelstein JA , Logvinenko T , Schuster MA . Incidence of Urinary Tract Infection Among Siblings of Children With Vesicoureteral Reflux . Acad Pediatr . 2016 ; 16 : 489 - 495 . 10.1016/j.acap.2015.11.003 PMC4867138 26589543

[47] 47. Arlen AM , Kirsch AJ , Leong T , Cooper CS . Validation of the ureteral diameter ratio for predicting early spontaneous resolution of primary vesicoureteral reflux . J Pediatr Urol . 2017 ; 13 : 383.e1 - 383.e6 . 10.1016/j.jpurol.2017.01.012 28256423

[48] 48. Jodal U , Smellie JM , Lax H , Hoyer PF . Ten-year results of randomized treatment of children with severe vesicoureteral reflux. Final report of the International Reflux Study in Children . Pediatr Nephrol . 2006 ; 21 : 785 - 92 . 10.1007/s00467-006-0063-0 16565873

[49] 49. Elder JS , Peters CA , Arant BS Jr , Ewalt DH , Hawtrey CE , Hurwitz RS , et al . Pediatric Vesicoureteral Reflux Guidelines Panel summary report on the management of primary vesicoureteral reflux in children . J Urol . 1997 ; 157 : 1846 - 51 . 9112544

[50] 50. Estrada CR Jr , Passerotti CC , Graham DA , Peters CA , Bauer SB , Diamond DA , et al . Nomograms for predicting annual resolution rate of primary vesicoureteral reflux: results from 2,462 children . J Urol . 2009 ; 182 : 1535 - 41 . 10.1016/j.juro.2009.06.053 19683762

[51] 51. Poulsen EU , Johannesen NL , Nielsen JB , Jørgensen TM , Andersen AJ . Vesico-ureteral reflux. II. The longterm outcome of kidney function in non-surgical treatment . Scand J Urol Nephrol Suppl . 1989 ; 125 : 29 - 34 . 2633315

[52] 52. Weiss R , Duckett J , Spitzer A . Results of a randomized clinical trial of medical versus surgical management of infants and children with grades III and IV primary vesicoureteral reflux (United States). The International Reflux Study in Children . J Urol . 1992 ; 148 ( 5 Pt 2 ): 1667 - 73 . 10.1016/s0022-5347(17)36998-7 1433585

[53] 53. [ No authors ]. Practice parameter: the diagnosis, treatment, and evaluation of the initial urinary tract infection in febrile infants and young children. American Academy of Pediatrics. Committee on Quality Improvement. Subcommittee on Urinary Tract Infection . Pediatrics . 1999 ; 103 ( 4 Pt 1 ): 843 - 52 . Erratum in: 2000 Jan;105(1 Pt 1):141. Pediatrics 1999;103(5 Pt 1):1052, 1999;104(1 Pt 1):118 . 10.1542/peds.103.4.843 10103321

[54] 54. Garin EH , Olavarria F , Garcia Nieto V , Valenciano B , Campos A , Young L . Clinical significance of primary vesicoureteral reflux and urinary antibiotic prophylaxis after acute pyelonephritis: a multicenter, randomized, controlled study . Pediatrics . 2006 ; 117 : 626 - 32 . 10.1542/peds.2005-1362 16510640

[55] 55. Montini G , Rigon L , Zucchetta P , Fregonese F , Toffolo A , Gobber D , et al . Prophylaxis after first febrile urinary tract infection in children? A multicenter, randomized, controlled, noninferiority trial . Pediatrics . 2008 ; 122 : 1064 - 71 . 10.1542/peds.2007-3770 18977988

[56] 56. Craig JC , Simpson JM , Williams GJ , Lowe A , Reynolds GJ , McTaggart SJ , et al . Prevention of Recurrent Urinary Tract Infection in Children with Vesicoureteric Reflux and Normal Renal Tracts (PRIVENT) Investigators. Antibiotic prophylaxis and recurrent urinary tract infection in children . N Engl J Med . 2009 ; 361 : 1748 - 59 . Erratum in: N Engl J Med. 2010;362:1250 . 10.1056/NEJMoa0902295 19864673

[57] 57. Brandström P , Jodal U , Sillén U , Hansson S . The Swedish reflux trial: review of a randomized, controlled trial in children with dilating vesicoureteral reflux . J Pediatr Urol . 2011 ; 7 : 594 - 600 . 10.1016/j.jpurol.2011.05.006 21807562

[58] 58. Carpenter MA , Hoberman A , Mattoo TK , Mathews R , Keren R , Chesney RW , et al . The RIVUR trial: profile and baseline clinical associations of children with vesicoureteral reflux . Pediatrics . 2013 ; 132 : e34 - 45 . 10.1542/peds.2012-2301 PMC3691529 23753091

[59] 59. de Bessa J Jr , de Carvalho Mrad FC , Mendes EF , Bessa MC , Paschoalin VP , Tiraboschi RB , et al . Antibiotic prophylaxis for prevention of febrile urinary tract infections in children with vesicoureteral reflux: a meta-analysis of randomized, controlled trials comparing dilated to nondilated vesicoureteral reflux . J Urol . 2015 ; 193 ( 5 Suppl ): 1772 - 7 . 10.1016/j.juro.2014.10.092 25817142

[60] 60. Weng H , Zeng XT . Antibiotic prophylaxis for prevention of febrile urinary tract infections in children with vesicoureteral reflux: a meta-analysis of randomized, controlled trials comparing dilated to nondilated vesicoureteral reflux . World J Urol . 2017 ; 35 : 847 - 8 . 10.1007/s00345-016-1935-y 27620895

[61] 61. Wong NC , Koyle MA , Braga LH . Continuous antibiotic prophylaxis in the setting of prenatal hydronephrosis and vesicoureteral reflux . Can Urol Assoc J . 2017 ; 11 ( 1-2 Suppl 1 ): S20 - S4 . 10.5489/cuaj.4387 PMC5332227 28265311

[62] 62. Wang HH , Gbadegesin RA , Foreman JW , Nagaraj SK , Wigfall DR , Wiener JS , et al . Efficacy of antibiotic prophylaxis in children with vesicoureteral reflux: systematic review and meta-analysis . J Urol . 2015 ; 193 : 963 - 9 . 10.1016/j.juro.2014.08.112 PMC4380260 25196653

[63] 63. Knudson MJ , Austin JC , McMillan ZM , Hawtrey CE , Cooper CS . Predictive factors of early spontaneous resolution in children with primary vesicoureteral reflux . J Urol . 2007 ; 178 ( 4 Pt 2 ): 1684 - 8 . 10.1016/j.juro.2007.03.161 17707023

[64] 64. Garcia-Roig M , Ridley DE , McCracken C , Arlen AM , Cooper CS , Kirsch AJ . Vesicoureteral Reflux Index: Predicting Primary Vesicoureteral Reflux Resolution in Children Diagnosed after Age 24 Months . J Urol . 2017 ; 197 : 1150 - 7 . 10.1016/j.juro.2016.12.008 27939835

[65] 65. Skoog SJ , Belman AB , Majd M . A nonsurgical approach to the management of primary vesicoureteral reflux . J Urol . 1987 ; 138 ( 4 Pt 2 ): 941 - 6 . 10.1016/s0022-5347(17)43465-3 3656575

[66] 66. Papachristou F , Printza N , Kavaki D , Koliakos G . The characteristics and outcome of primary vesicoureteric reflux diagnosed in the first year of life . Int J Clin Pract . 2006 ; 60 : 829 - 34 . 10.1111/j.1742-1241.2006.00859.x 16704677

[67] 67. Schwab CW Jr , Wu HY , Selman H , Smith GH , Snyder HM 3rd , Canning DA . Spontaneous resolution of vesicoureteral reflux: a 15-year perspective . J Urol . 2002 ; 168 : 2594 - 9 . 10.1016/S0022-5347(05)64225-5 12441993

[68] 68. Yeung CK , Sreedhar B , Sihoe JD , Sit FK . Renal and bladder functional status at diagnosis as predictive factors for the outcome of primary vesicoureteral reflux in children . J Urol . 2006 ; 176 : 1152 - 6 . 10.1016/j.juro.2006.04.053 16890714

[69] 69. Nepple KG , Knudson MJ , Austin JC , Cooper CS . Abnormal renal scans and decreased early resolution of low grade vesicoureteral reflux . J Urol . 2008 ; 180 ( 4 Suppl ): 1643 - 7 ; discussion 1647 . 10.1016/j.juro.2008.03.102 18715588

[70] 70. Arlen AM , Garcia-Roig M , Weiss AD , Leong T , Cooper CS , Kirsch AJ . Vesicoureteral Reflux Index: 2-Institution Analysis and Validation . J Urol . 2016 ; 195 ( 4 Pt 2 ): 1294 - 9 . 10.1016/j.juro.2015.03.094 25813448

[71] 71. Brandström P , Esbjörner E , Herthelius M , Swerkersson S , Jodal U , Hansson S . The Swedish reflux trial in children: III. Urinary tract infection pattern . J Urol . 2010 ; 184 : 286 - 91 . 10.1016/j.juro.2010.01.061 20488494

[72] 72. Silva JM , Diniz JS , Lima EM , Vergara RM , Oliveira EA . Predictive factors of resolution of primary vesico-ureteric reflux: a multivariate analysis . BJU Int . 2006 ; 97 : 1063 - 8 . 10.1111/j.1464-410X.2006.06064.x 16643493

[73] 73. Arlen AM , Alexander SE , Wald M , Cooper CS . Computer model predicting breakthrough febrile urinary tract infection in children with primary vesicoureteral reflux . J Pediatr Urol . 2016 ; 12 : 288.e1 - 288.e5 . 10.1016/j.jpurol.2016.03.005 27072485

[74] 74. Austin JC , Cooper CS . Vesicoureteral reflux: who benefits from correction . Urol Clin North Am . 2010 ; 37 : 243 - 52 . 10.1016/j.ucl.2010.03.012 20569802

[75] 75. Routh JC , Bogaert GA , Kaefer M , Manzoni G , Park JM , Retik AB , et al . Vesicoureteral reflux: current trends in diagnosis, screening, and treatment . Eur Urol . 2012 ; 61 : 773 - 82 . 10.1016/j.eururo.2012.01.002 22264440

[76] 76. Hsieh MH , Madden-Fuentes RJ , Bayne A , Munch E , Wildenfels P , Alexander SJ , et al . Cross-sectional evaluation of parental decision making factors for vesicoureteral reflux management in children . J Urol . 2010 ; 184 ( 4 Suppl ): 1589 - 93 . 10.1016/j.juro.2010.03.083 20728107

[77] 77. Sung J , Skoog S . Surgical management of vesicoureteral reflux in children . Pediatr Nephrol . 2012 ; 27 : 551 - 61 . 10.1007/s00467-011-1933-7 PMC3288369 21695451

[78] 78. Yeung CK , Chowdhary SK , Sreedhar B . Minimally Invasive Management for Vesicoureteral Reflux in Infants and Young Children . Clin Perinatol . 2017 ; 44 : 835 - 49 . 10.1016/j.clp.2017.08.008 29127964

[79] 79. Roihuvuo-Leskinen HM , Vainio MI , Niskanen KM , Lahdes-Vasama TT . Pregnancies in women with childhood vesicoureteral reflux . Acta Obstet Gynecol Scand . 2015 ; 94 : 847 - 51 . 10.1111/aogs.12664 25912311

[80] 80. Upadhyay J , McLorie GA , Bolduc S , Bägli DJ , Khoury AE , Farhat W . Natural history of neonatal reflux associated with prenatal hydronephrosis: long-term results of a prospective study . J Urol . 2003 ; 169 : 1837 - 41 ; discussion 1841; author reply 1841 . 10.1097/01.ju.0000062440.92454.cf 12686858

[81] 81. Farhat W , McLorie G , Geary D , Capolicchio G , Bägli D , Merguerian P , et al . The natural history of neonatal vesicoureteral reflux associated with antenatal hydronephrosis . J Urol . 2000 ; 164 ( 3 Pt 2 ): 1057 - 60 . 10.1097/00005392-200009020-00033 10958740

[82] 82. Kim SW , Lee YS , Han SW . Endoscopic injection therapy . Investig Clin Urol . 2017 ; 58 ( Suppl 1 ): S38 - S45 . 10.4111/icu.2017.58.S1.S38 PMC5468263 28612059

[83] 83. Fuentes S , Gómez-Fraile A , Carrillo-Arroyo I , Tordable-Ojeda C , Cabezalí-Barbancho D . Endoscopic Treatment of Vesicoureteral Reflux in Infants. Can We Do It and Should We Do It? Urology . 2017 ; 10 : 196 - 200 . 10.1016/j.urology.2017.08.005 28818534

[84] 84. Elder JS , Shah MB , Batiste LR , Eaddy M . Part 3: Endoscopic injection versus antibiotic prophylaxis in the reduction of urinary tract infections in patients with vesicoureteral reflux . Curr Med Res Opin . 2007 ; 23 ( Suppl 4 ): S15 - 20 . 10.1185/030079907X226230 17931480

[85] 85. Esposito C , Escolino M , Lopez M , Farina A , Cerulo M , Savanelli A , et al . Surgical Management of Pediatric Vesicoureteral Reflux: A Comparative Study Between Endoscopic, Laparoscopic, and Open Surgery . J Laparoendosc Adv Surg Tech A . 2016 ; 26 : 574 - 80 . 10.1089/lap.2016.0055 27284903

[86] 86. Solomon LZ , Birch BR , Cooper AJ , Davies CL , Holmes SA . Nonhomologous bioinjectable materials in urology: ‘size matters’? BJU Int . 2000 ; 85 : 641 - 5 . 10.1046/j.1464-410x.2000.00521.x 10759657

[87] 87. Chertin B , Puri P . Endoscopic management of vesicoureteral reflux: does it stand the test of time? Eur Urol . 2002 ; 42 : 598 - 606 . 10.1016/s0302-2838(02)00447-5 12477657

[88] 88. Chertin B , Kocherov S , Chertin L , Natsheh A , Farkas A , Shenfeld OZ , et al . Endoscopic bulking materials for the treatment of vesicoureteral reflux: a review of our 20 years of experience and review of the literature . Adv Urol . 2011 ; 2011 : 309626 . 10.1155/2011/309626 PMC3095422 21603212

[89] 89. Kirsch AJ , Perez-Brayfield MR , Scherz HC . Minimally invasive treatment of vesicoureteral reflux with endoscopic injection of dextranomer/hyaluronic acid copolymer: the Children’s Hospitals of Atlanta experience . J Urol . 2003 ; 170 : 211 - 5 . 10.1097/01.ju.0000072523.43060.a0 12796692

[90] 90. Lee EK , Gatti JM , Demarco RT , Murphy JP . Long-term followup of dextranomer/hyaluronic acid injection for vesicoureteral reflux: late failure warrants continued followup . J Urol . 2009 ; 181 : 1869 - 74 ; discussion 1874-5 . 10.1016/j.juro.2008.12.005 19233403

[91] 91. Ormaechea M , Ruiz E , Denes E , Gimenez F , Dénes FT , Moldes J , et al . New tissue bulking agent (polyacrylate polyalcohol) for treating vesicoureteral reflux: preliminary results in children . J Urol . 2010 ; 183 : 714 - 7 . 10.1016/j.juro.2009.10.047 20022037

[92] 92. Şencan A , Yıldırım H , Özkan KU , Uçan B , Karkıner A , Hoşgör M . Late ureteral obstruction after endoscopic treatment of vesicoureteral reflux with polyacrylate polyalcohol copolymer . Urology . 2014 ; 84 : 1188 - 93 . 10.1016/j.urology.2014.07.030 25443932

[93] 93. Karakus SC , User İR , Kılıc BD , Akçaer V , Ceylan H , Ozokutan BH . The comparison of dextranomer/hyaluronic acid and polyacrylate-polyalcohol copolymers in endoscopic treatment of vesicoureteral reflux . J Pediatr Surg . 2016 ; 51 : 1496 - 500 . 10.1016/j.jpedsurg.2016.02.092 27061353

[94] 94. Ozkuvanci U , Donmez MI , Ozgor F , Erbin A , Pasin Ö , Muslumanoglu AY . Durasphere® EXP: a non-biodegradable agent for treatment of primary Vesico-Ureteral reflux in children . Int Braz J Urol . 2018 ; 44 : 585 - 90 . 10.1590/S1677-5538.IBJU.2017.0514 PMC5996799 29522294

[95] 95. Stenberg A , Läckgren G . Treatment of vesicoureteral reflux in children using stabilized non-animal hyaluronic acid/dextranomer gel (NASHA/DX): a long-term observational study . J Pediatr Urol . 2007 ; 3 : 80 - 5 . 10.1016/j.jpurol.2006.08.001 18947708

[96] 96. Yap TL , Chen Y , Nah SA , Ong CC , Jacobsen A , Low Y . STING versus HIT technique of endoscopic treatment for vesicoureteral reflux: A systematic review and meta-analysis . J Pediatr Surg . 2016 ; 51 : 2015 - 20 . 10.1016/j.jpedsurg.2016.09.028 27773360

[97] 97. Kirsch AJ , Arlen AM . Evaluation of new Deflux administration techniques: intraureteric HIT and Double HIT for the endoscopic correction of vesicoureteral reflux . Expert Rev Med Devices . 2014 ; 11 : 439 - 46 . 10.1586/17434440.2014.929491 24931132

[98] 98. Kirsch AJ , Perez-Brayfield M , Smith EA , Scherz HC . The modified sting procedure to correct vesicoureteral reflux: improved results with submucosal implantation within the intramural ureter . J Urol . 2004 ; 171 ( 6 Pt 1 ): 2413 - 6 . 10.1097/01.ju.0000127754.79866.7f 15126864

[99] 99. Leung L , Chan IHY , Chung PHY , Lan LCL , Tam PKH , Wong KKY . Endoscopic injection for primary vesicoureteric reflux: Predictors of resolution and long term efficacy . J Pediatr Surg . 2017 ; 52 : 2066 - 9 . 10.1016/j.jpedsurg.2017.08.033 28927982

[100] 100. Lorenzo AJ , Pippi Salle JL , Barroso U , Cook A , Grober E , Wallis MC , et al . What are the most powerful determinants of endoscopic vesicoureteral reflux correction? Multivariate analysis of a single institution experience during 6 years . J Urol . 2006 ; 176 ( 4 Pt 2 ): 1851 - 5 . 10.1016/S0022-5347(06)00599-4 16945671

[101] 101. Higham-Kessler J , Reinert SE , Snodgrass WT , Hensle TW , Koyle MA , Hurwitz RS , et al . A review of failures of endoscopic treatment of vesicoureteral reflux with dextranomer microspheres . J Urol . 2007 ; 177 : 710 - 4 ; discussion 714-5 . 10.1016/j.juro.2006.09.082 17222662

[102] 102. Rubenwolf PC , Ebert AK , Ruemmele P , Rösch WH . Delayed-onset ureteral obstruction after endoscopic dextranomer/hyaluronic acid copolymer (Deflux) injection for treatment of vesicoureteral reflux in children: a case series . Urology . 2013 ; 81 : 659 - 62 . 10.1016/j.urology.2012.11.044 23452811

[103] 103. Hutch JA . Vesico-ureteral reflux in the paraplegic: cause and correction . J Urol . 1952 ; 68 : 457 - 69 . 10.1016/S0022-5347(17)68223-5 14955874

[104] 104. Riedmiller H , Gerharz EW . Antireflux surgery: Lich-Gregoir extravesical ureteric tunnelling . BJU Int . 2008 ; 101 : 1467 - 82 . 10.1111/j.1464-410X.2008.07683.x 18454801

[105] 105. Glenn JF , Anderson EE . Distal tunnel ureteral reimplantation . J Urol . 1967 ; 97 : 623 - 6 . 10.1016/S0022-5347(17)63089-1 6022427

[106] 106. Steffens J , Stark E , Haben B , Treiyer A . Politano-Leadbetter ureteric reimplantation . BJU Int . 2006 ; 98 : 695 - 712 . 10.1111/j.1464-410X.2006.06407.x 16925783

[107] 107. Castellán M , García Mérida M , Gosálbez R . [ Transitory urinary retention after simultaneous bilateral extravesical ureteral reimplantation ]. Arch Esp Urol . 2008 ; 61 : 316 - 9 . 10.4321/s0004-06142008000200031 18491753

[108] 108. Silay MS , Turan T , Kayalı Y , Başıbüyük İ , Gunaydin B , Caskurlu T , et al . Comparison of intravesical (Cohen) and extravesical (Lich-Gregoir) ureteroneocystostomy in the treatment of unilateral primary vesicoureteric reflux in children . J Pediatr Urol . 2018 ; 14 : 65.e1 - 65.e4 . 10.1016/j.jpurol.2017.09.014 29146303

[109] 109. Carrillo Arroyo I , Fuentes Carretero S , Gómez Fraile A , Morante Valverde R , Tordable Ojeda C , Cabezalí Barbancho D . Technical challenges of endoscopic treatment for vesicoureteral reflux after Cohen reimplantation . Actas Urol Esp . 2019 ; 43 : 384 - 8 . 10.1016/j.acuro.2019.02.004 31103394

[110] 110. Heidenreich A , Ozgur E , Becker T , Haupt G . Surgical management of vesicoureteral reflux in pediatric patients . World J Urol . 2004 ; 22 : 96 - 106 . 10.1007/s00345-004-0408-x 15221260

[111] 111. Soulier V , Scalabre AL , Lopez M , Li CY , Thach S , Vermersch S , et al . Laparoscopic vesico-ureteral reimplantation with Lich-Gregoir approach in children: medium term results of 159 renal units in 117 children . World J Urol . 2017 ; 35 : 1791 - 8 . 10.1007/s00345-017-2064-y 28638940

[112] 112. Silay MS , Baek M , Koh CJ . Robot-Assisted Laparoscopic Extravesical Ureteral Reimplantation in Children: Top-Down Suturing Technique Without Stent Placement . J Endourol . 2015 ; 29 : 864 - 6 . 10.1089/end.2014.0815 25674670

[113] 113. Arlen AM , Broderick KM , Travers C , Smith EA , Elmore JM , Kirsch AJ . Outcomes of complex robot-assisted extravesical ureteral reimplantation in the pediatric population . J Pediatr Urol . 2016 ; 12 : 169.e1 - 6 . 10.1016/j.jpurol.2015.11.007 26747012

